# The Serum Concentrations of Chemokine CXCL12 and Its Specific Receptor CXCR4 in Patients with Esophageal Cancer

**DOI:** 10.1155/2016/7963895

**Published:** 2016-03-03

**Authors:** Marta Łukaszewicz-Zając, Barbara Mroczko, Mirosław Kozłowski, Maciej Szmitkowski

**Affiliations:** ^1^Department of Biochemical Diagnostics, Medical University, Waszyngtona 15A, 15-269 Białystok, Poland; ^2^Department of Biochemical Diagnostics, University Hospital, Waszyngtona 15A, 15-269 Białystok, Poland; ^3^Department of Neurodegeneration Diagnostics, Medical University, Waszyngtona 15A, 15-269 Białystok, Poland; ^4^Department of Thoracic Surgery, Medical University, Marii Skłodowskiej-Curie 24A, 15-276 Białystok, Poland

## Abstract

*Objectives.* Recent investigations have suggested that upregulated levels of inflammatory biomarkers, such as chemokines, may be associated with development of many malignancies, including esophageal cancer (EC). Based on our knowledge, this study is the first to assess the serum concentration of chemokine CXCL12 and its specific receptor CXCR4 in the diagnosis of EC patients.* Material and Methods.* The present study included 79 subjects: 49 patients with EC and 30 healthy volunteers. The serum concentrations of CXCL12 and CXCR4 and classical tumor markers such as carcinoembryonal antigen (CEA) and squamous cell cancer antigen (SCC-Ag) were measured using immunoenzyme assays, while C-reactive protein (CRP) levels were assessed by immunoturbidimetric method. Moreover, diagnostic criteria of all proteins tested and the survival of EC patients were assessed.* Results.* The serum concentrations of CXCL12 were significantly higher, while those of its receptor CXCR4 were significantly lower in EC patients compared to healthy controls. The diagnostic sensitivity, negative predictive value, and accuracy of CXCR4 were the highest among all analyzed proteins and increased for combined analysis with classical tumor markers and CRP levels.* Conclusion.* Our findings suggest that serum CXCR4 may improve the diagnosis of EC patients, especially in combination with classical tumor markers.

## 1. Introduction

Chemokines are a family of soluble chemotactic cytokines that bind to their cognate G-protein coupled receptors. These proteins regulate leukocytes' migration, adhesion, or chemotaxis and therefore play important roles in inflammation and tissue injury as well as in the development of malignant diseases, including the tumors of esophagus [1–4]. Esophageal cancer (EC) is still one of the deadliest neoplasms worldwide, characterized by the aggressive behavior and late stage of diagnosis of EC patients. This neoplasm is the eight most common malignant disease and the sixth cause of cancer-related deaths worldwide [5]. There are two major histological types of EC—adenocarcinoma of esophagus (AC) and esophageal squamous cell carcinoma (ESCC). The epidemiology and risk factors of both types of EC were proved to be different; thus they might affect the incidence and distribution of these tumors [5–9]. It has been indicated that in developed countries the adenocarcinoma of esophagus became the most common type of EC, due to increased gastroesophageal reflux disease (GERD) and obesity prevalence [6–10]. However, the major histologic type in the world is still ESCC, especially in Africa and the Middle East. The most frequent risk factors for ESCC are abuse of alcohol and tobacco, as well as nutritional deficits [5]. It has been indicated that Barrett's esophagus was indicated to be the preneoplastic lesion for AC, whereas squamous dysplasia is the precursor lesion of ESCC [11].

The prognosis of EC patients' survival remains unfavorable, due to lack of early symptoms of disease and late EC patients' diagnosis. Endoscopic ultrasonography and computed tomography are routine methods of diagnosis of EC patients; however they have a limited usefulness in the early detection of this malignancy [12, 13]. In addition, the measurements of biochemical tumor markers concentrations for EC, such as carcinoembryonal antigen (CEA) and squamous cell cancer antigen (SCC-Ag), have been useful in the routine diagnosis and follow-up of patients with this malignancy, although their diagnostic sensitivity and specificity are still not satisfactory [14]. Thus, new diagnostic and prognostic biomarkers are crucial to improve diagnosis and survival of patients with EC.

The C-X-C motif chemokine 12 (CXCL12), also known as stromal cell-derived factor 1 (SDF-1), interacts with its specific receptor (CXCR4) to form a coupled molecular pair to pass information about the growth, adhesion, and migration of leucocytes. However, it has been shown that CXCL12/CXCR4 might be expressed also in many tumor cells [15, 16]. Therefore, there is increasing evidence that CXCL12 and its specific receptor CXCR4 play an important role in many steps of tumor progression, such as invasion, migration, and proliferation of malignant cells. Some authors indicated that CXCL12 and its receptor CXCR4 were expressed in EC tissue and significantly correlated with invasion, angiogenesis, lymph node, and metastasis as well as prognosis of EC patients' survival [15–19]. However, according to our knowledge, our present study is the first to assess the serum concentrations of chemokine CXCL12 in relation to its specific receptor CXCR4 and classical tumor markers for EC (CEA and SCC-Ag) as well as marker of inflammatory states—C-reactive protein (CRP). The association between serum concentrations of all proteins tested and histological types of EC, such as AC and ESCC, was also revealed. Moreover, the relationship between serum concentrations of CXCL12/CXCR4 and clinicopathological parameters of tumor as well as the EC patients' survival was presented. In addition, the diagnostic criteria including diagnostic sensitivity and specificity, accuracy, and predictive values for negative (NPV) and positive (PPV) results as well as the areas under the ROC curve (AUC) for all the proteins tested were also calculated.

## 2. Methods and Materials

The study included 49 EC subjects (aged 44–80 years, 8 women and 41 men) diagnosed in the Department of Thoracic Surgery (University Hospital of Białystok, Poland). Among the 49 patients with EC, 25 patients suffered from ESCC, while in 24 patients AC was diagnosed. The microscopic examination of material obtained during biopsy and/or surgery was used in the clinical diagnosis of EC patients. All the patients with EC were staged according to the TNM (tumor-nodules-metastases) classification, presented by the International Union Against Cancer (UICC) [20]. The characteristic of EC patients was presented in Table 1. Control group consists of 30 healthy volunteers (16 women and 14 men, aged 22–66 years).

The study group, analyzed in this paper, were divided into following groups, depending on stage of tumor (TNM), depth of tumor invasion (T factor), the presence of lymph node (N factor) and distant metastases (M factor), and histological grade (G factor) as well as resectability of tumor. The patients gave informed consent and the present project was approved by the Local Ethics Committee (R-I-002/42/2015) of Medical University of Białystok (Poland).

The blood samples from the patients were obtained before the treatment. The serum samples were stored at −80°C until the analyses. The serum CXCL12 and CXCR4 levels were measured using ELISA (enzyme-linked immunosorbent assay) kits (EIAab, Wulhan, China) based on the instructions from manufacturer. The intra-assay coefficient of variation (CV) for CXCL12 is presented by the manufacturer as ≤7.6%, while CV for CXCR4 was indicated by the manufacturer to be ≤7.8%. The concentrations of serum CEA and SCC-Ag were assessed using chemiluminescent microparticle immunoassay (CMIA) kits (Abbott, United States of America). The CV for CEA was presented by the manufacturer to be 4.9% at a mean concentration of 2.2 ng/mL (SD = 0.11 ng/mL), while the intra-assay CV% is referred to by the manufacturer of the assay kit as 4.3% at SCC-Ag mean concentration of 1.97 ng/mL, SD = 0.085. Serum CRP levels were measured using immunoturbidimetric C-REACTIVE PROTEIN assay kit (Abbott, United States of America), according to the manufacturer's instruction.

The reference cut-off values of CXCL12 (1.44 ng/mL) and CXCR4 (0.79 ng/mL) correspond to the highest accuracy (minimal false-negative and false-positive results), while cut-off values for CEA (4 ng/mL), SCC-Ag (2 ng/mL), and CRP (5.75 ng/mL) (the 95th percentile) were established previously in our department [21–24].

### 2.1. Statistical Analysis

The concentrations of all proteins tested did not follow a normal distribution in the preliminary statistical analysis (*χ* ^2^-test), and thus nonparametric statistical analyses were employed. The Mann-Whitney test was used to compare two groups, whereas the Kruskal-Wallis test was performed for three or more groups. Additionally, the post hoc Dwass-Steele-Critchlow-Fligner test was performed to analyze which groups were different, if the significant differences were assessed. Moreover, the diagnostic criteria such as diagnostic sensitivity and specificity, accuracy, and predictive value for negative (NPV) and positive (PPV) results of CXCL12, CXCR4, CEA, SCC-Ag, and CRP were assessed. STATISTICA 5.1 PL (StatSoft Inc., USA) was used for statistical analysis. Moreover, the MedCalc statistical software (Mariakerke, Belgium) and Microsoft Office Excel were employed for the diagnostic criteria. In addition, for the analysis of survival curves the Kaplan and Meier test was employed. The log-rank test was used for the univariate analyses of survival, while the Cox proportional hazards model was employed for multivariate analyses. The differences were considered to be statically significant when *p* < 0.05. Our findings were presented as median and range.

## 3. Results

The medians and ranges of the serum concentrations of chemokine CXCL12 and its receptor CXCR4 as well as classical tumor markers (CEA and SCC-Ag) and marker of inflammatory states (CRP) in EC patients and healthy volunteers were presented in Table 2. The serum CXCL12 levels were significantly higher (*p* = 0.044), while its receptor CXCR4 was significantly lower (*p* = 0.031) in EC patients compared to healthy controls. The concentrations of classical tumor markers as well as CRP were found to be higher in EC patients than in control group (Table 2).

If we analyze the relationship between serum levels of proteins tested and histological type of EC, the serum concentrations of CXCL12 were significantly higher in patients with ESCC than in control group, similarly to classical tumor markers and CRP. The significant differences between AC subgroup and healthy controls were demonstrated for CXCR4, CRP, and CEA concentrations (Table 2). In addition, the highest serum concentrations of CXCL12 and CXCR4 were found in III stage of EC, similarly to SCC-Ag levels; however these differences were not statistically significant (data not shown).

If we consider the association between the concentrations of proteins tested and clinicopathological parameters of EC, the CXCL12 and its receptor levels increased with the depth of tumor invasion and the presence of lymph node metastasis and were the highest in T4 and N1 subgroups, similarly to SCC-Ag concentrations. However, the differences between analyzed subgroups (T1 + 2, T3 and T4) were significant only for CRP levels (*p* = 0.002) (data not shown). Additionally, the serum concentrations of chemokine CXCL12 and its receptor were the highest in poorly differentiated tumors (G3), similar to the CRP and SCC-Ag concentrations. In our study, we also indicated that the concentrations of CXCL12 and CXCR4 were higher in patients with resectable tumors compared to those with nonresectable EC, similarly to classical tumor markers (data not shown).

Univariate Cox's proportional hazards models revealed that the histological types of EC (ESCC versus AC) (*p* = 0.029), tumor stage (*p* = 0.001), tumor size (*p* = 0.001), the presence of distant metastases (*p* < 0.001), resectability of tumor (*p* = 0.003), tumor length (*p* = 0.012), and the CRP levels (*p* = 0.024) were the significant factors affecting the overall survival. Multivariate regression analyses with Cox's proportional hazards model have demonstrated that only the resectability of tumor was found to be independent prognostic factor for the EC patients' survival.

The percentages of elevated results (diagnostic sensitivity) of CXCR4 (80%) were higher than those of CXCL12 (47%) and much higher than classical tumor markers—CEA (22%) and SCC-Ag (14%) as well as CRP (57%). In addition, the frequency of increased concentrations was the highest for CXCL12 with CXCR4 levels in combination (94%) and improved the diagnostic sensitivity of combined measurement of classical tumor markers—CEA with SCC-Ag (33%) (Table 3). The diagnostic specificity for CXCL12 levels (80%) was lower than that for classical tumor markers and CRP, similarly to positive predictive value (PPV). However, the negative predictive value for CXCR4 (63%) was the highest among all analyzed proteins. The highest NPV was observed for combined use of CXCR4 with CRP (80%). The diagnostic accuracy of CXCR4 (71%) was similar to CRP (72%), much higher in comparison to CXCL12 (59%) and classical tumor markers (CEA: 49%, SCC-Ag: 46%), and increased to 77% in combined measurement with CRP (Table 3). The Youden Index (YI) is used to assess the effectiveness of a diagnostic marker and gives equal weight to sensitivity and specificity for the biomarker concentrations. The highest YI among all proteins tested was observed for the measurement of CRP.

**Figure 1:**
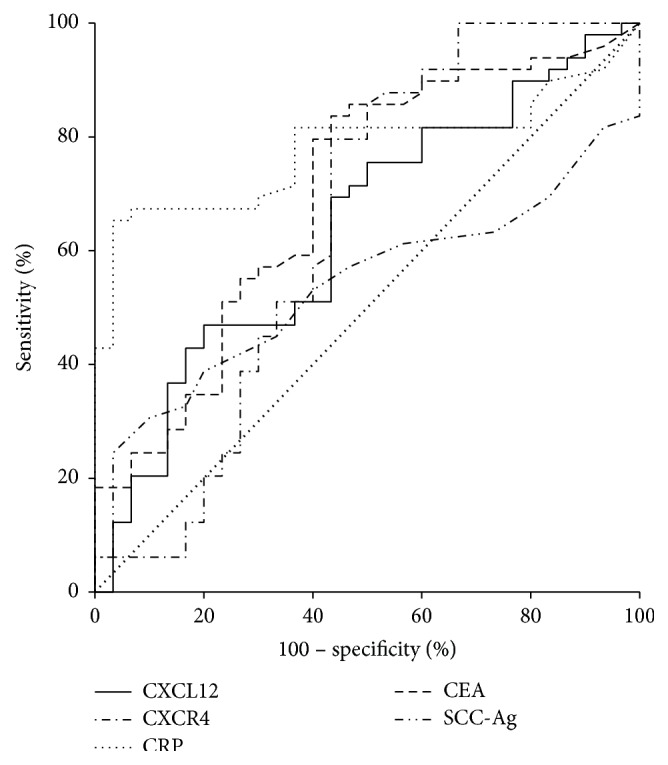
Areas under ROC curves (AUC) for CXCL12 (AUC = 0.6354; *p* = 0.0371), CXCR4 (AUC = 0.6456; *p* = 0.0450), and classical tumor markers—CEA (AUC = 0.6973; *p* = 0.0016) and SCC-Ag (AUC = 0.5384; *p* = 0.552) as well as CRP (AUC = 0.7779; *p* < 0.001) in esophageal cancer patients.

The area under the ROC curve (AUC) indicates the clinical usefulness of the proteins tested in the diagnosis of patients. The AUC of CXCR4 (0.6456; *p* = 0.0450) was similar to AUC for CXCL12 (0.6354; *p* = 0.0371) and CEA (0.6973; *p* = 0.0016) and higher than that for SCC-Ag (0.5384; *p* = 0.5522) in the diagnosis of EC patients. The highest AUC was calculated for serum CRP levels (0.7779; *p* < 0.001) (Figure 1).

## 4. Discussion

The main significance of chemokines and their receptors in tumor development is facilitating the invasion and metastasis of malignant cells, including adherence, proliferation, extravasation from blood vessels, angiogenesis, and protection from the host response [1–5]. Some authors suggested the potential role of selected chemokines and/or their receptors in the development of various types of tumors, including EC [15–19]. These neoplasms are characterized by poor prognosis of patients' survival. Well-established, biochemical tumor markers for this malignancy, such as CEA and SCC-Ag, are commonly used in routine practice; however their sensitivity is unsatisfactory. Some researchers suggested the role of chemokine CXCL12 and its receptor CXCR4 in the development of EC, but these studies were performed only on EC tissue, using mostly immunohistochemistry technique. To our knowledge, this is the first report assessing the concentrations of CXCL12 and CXCR4 in the serum of patients with EC in relation to clinicopathological characteristic of tumor as well as diagnostic and prognostic potential of proteins tested in comparison to classical tumor markers and marker of inflammation (CRP). The present paper is continuation of our previous findings concerning the role of selected inflammatory proteins, such as C-reactive protein (CRP), interleukin 6 (IL-6), hematopoietic cytokines (HGFs), and metalloproteinases (MMPs) as tumor markers for EC [21–24].

In our present paper, the serum concentrations of CXCL12 were significantly higher, while CXCR4 was significantly lower in EC patients compared to healthy controls, while the levels of classical tumor markers as well as CRP were found to be also higher in EC patients than in control group. We suggest that increased concentrations of CXCL12 and low levels of its specific receptor in EC might be results of improved ability of CXCR4 receptors to bind the higher amount of CXCL12 in cancer patients. In addition, the highest serum concentrations of CXCL12 and CXCR4 were found in III stage of EC, similarly to SCC-Ag levels. It may be suggested that upregulation of CXCL12 and its specific receptor levels with increase in III stage of EC might be a result of cancer ability to spread [25]. Other authors also indicated that high CXCL12 levels may be related with a tendency to metastasis of cancer cells, while the low CXCL12 expression exists in the site of primary EC [16, 17].

If we consider the association between serum levels of proteins tested and histological type of EC, the concentrations of CXCL12 were significantly higher in patients with ESCC than in control group, similar to classical tumor markers and CRP. The statistical differences between AC subgroup and healthy controls were demonstrated for CXCR4, CRP, and CEA concentrations. In our previous findings we assessed the diagnostic usefulness of other cytokines in the sera of EC patients [22, 23]. We revealed that the serum levels of interleukin 6 (IL-6) and macrophage stimulating factor (M-CSF) were also significantly higher in ESCC patients than in healthy controls [22, 23].

The relationship between the concentrations of proteins tested and clinicopathological parameters of EC was also analyzed in our present study. We indicated that the CXCL12 and its receptor levels were the highest in T4 and N1 subgroups, similar to CRP and SCC-Ag concentrations. Other authors revealed that there were no correlations between CXCR4 [15] or CXCL12 [16] levels and clinicopathological variables of EC. On the contrary to our results, some authors concluded that the CXCL12 and its receptor levels depended on the amount of cancer cells; however these findings were performed on EC tissue using immunohistochemistry technique [15–18]. The positive CXCL12 expression was significantly correlated with lymph node metastasis, tumor stage, and lymphatic invasion [15], whereas the CXCR4 expression was significantly higher in the ESCC patients with lymph node metastasis and T3 stage of tumor than in group without lymph node metastasis and patients with T1-T2 stages [15–18]. The authors conclude that CXCR4 plays an important role in the progression of EC [18]. In our present data we demonstrated that serum concentrations of chemokine CXCL12 and its receptor were the highest in poorly differentiated tumors, similarly to the CRP and SCC-Ag levels. We presented similar results in our previous studies, where we indicated that the serum levels of IL-6 and M-CSF were also the highest in undifferentiated EC (G3) when compared with well and moderately differentiated tumors (G1 and G2), and these differences were also not significant [22, 23].

Univariate Cox's proportional hazards models failed to establish that CXCL12 and its receptor might be the significant factors affecting the overall survival. Multivariate regression analysis revealed that none of the proteins analyzed in the study were found to be independent prognostic factor for the survival of EC patients. Similar results were presented by Gockel et al., who also did not indicate the correlation between CXCR4 and prognosis for ESCC and AC patients [26]. Opposite results were demonstrated by other studies using univariate analysis, where the overall and disease-free survival rates were significantly lower in patients with positive CXCL12 expression compared to those with negative CXCL12 immunoreactivity. The investigators concluded that CXCL12 expression is an important predictor of lymph node metastasis and poor prognosis of ESCC patients' survival [15]. In addition, higher expression of CXCR4 was found to be negative independent prognostic factor of ESCC patients' survival [16]. The discrepancy between our report and findings of other authors may be a result of different biological material and method used in analyses. In our research, the serum samples from EC patients were employed to measure the concentrations of all proteins tested by ELISA method, while other investigators performed their studies on tissue samples to assess the expression of these proteins mostly in immunohistochemistry technique [15–18].

Diagnostic criteria, such as diagnostic sensitivity and specificity as well as AUC of biomarkers tested, are crucial for the detection of malignant disease and follow-up and therapy selection [27]. In our study, we indicated that diagnostic sensitivity of CXCR4 was the highest among all proteins tested and increased to 94% for combined analysis of CXCL12 with CXCR4 levels and was higher than diagnostic sensitivity of CEA with SCC-Ag in combination. Similar observations were established for the NPV for CXCR4 levels, which was the highest among all analyzed proteins and increased for combined use of CXCR4 with CRP to 80%. The diagnostic accuracy of CXCR4 was similar to CRP, much higher than that for CXCL12 and classical tumor markers and increased to 77% in combination with CRP. We obtained similar results in our previous studies, where we assessed the diagnostic usefulness of other cytokines in EC patients [22, 23]. We observed that the diagnostic sensitivity of IL-6 and M-CSF was also higher than those of CEA and SCC-Ag and increased in combined use with classical tumor markers for EC [22, 23]. Moreover, in our present paper, the AUC for CXCR4 was similar to AUC for CXCL12 and CEA and higher than that for SCC-Ag in the diagnosis of EC patients. These findings were in line with our previous results, where the AUC of IL-6 was larger than that for both classical tumor markers, while AUC for M-CSF was found to be higher than CEA in EC [22, 23]. It has been shown that the assessment of single biomarker is not accurate enough to be used as diagnostics tool due to their not-specific nature, while the measurement of selected inflammatory proteins especially with CXCR4 in combination with classical tumor markers may improve the diagnosis of EC patients.

The alterations between our present results and the findings of other authors suggest that the role of CXCL12/CXCR4 pathway in EC has not been fully evaluated. Moreover, our data was the first that presented the serum concentrations of chemokine CXCL12 and its specific receptor in EC patients, whereas other authors presented the expression of these proteins in EC tissue [28–30]. Therefore, our findings need to be confirmed in future studies performed on larger population of EC patients.

## 5. Conclusion

In conclusion, our finding revealed that serum CXCL12 was significantly related to the levels of its specific receptor CXCR4; thus the CXCL12/CXCR4 axis may play a role in the development of EC. The serum concentrations of CXCL12 were significantly higher, while its specific receptor CXCR4 was significantly lower in EC patients compared to healthy controls. The diagnostic sensitivity, negative predictive value, and accuracy of CXCR4 were the highest among all analyzed proteins and increased for combined analysis with classical tumor markers and CRP levels. Based on our present results, we suggest that serum CXCR4 may improve the diagnosis of EC patients, especially in combination with classical tumor markers. However, due to nonspecific nature of chemokines, these findings need to be confirmed in future studies performed on larger population of patients with EC.

## Figures and Tables

**Table 1:** Characteristics of esophageal cancer patients.

Variable tested		Number of patients
Group	Esophageal cancer	49

Gender	Male	41
	Female	8

Type of cancer	Adenocarcinoma	24
	Planoepitheliale	25

TNM stage (2)	I + IIa + IIb	10
	III	32
	IV	7

Depth of tumor invasion (T factor)	T1 + T2	8
	T3	29
	T4	12

Nodal involvement (N factor)	N0	11
	N1	38

Distant metastases (M factor)	M0	42
	M1	7

Differentiation of tumor	Well differentiated—G1	14
	Moderately differentiated—G2	18
	Undifferentiated—G3	17

Resection	R0—microscopically complete	23
	Other	26

Survival of patients	Died of cancer	22
	Alive	27

**Table 2:** Serum levels of proteins tested in patients with esophageal cancer in comparison with healthy controls.

Group tested		CXCL12 (ng/mL)	CXCR4 (ng/mL)	CEA (ng/mL)	SCC-Ag (ng/mL)	CRP (ng/mL)
Control group (*n* = 30)	Median (range)	**0.865** (0.105–4.297)	**0.932** (0.079–2.879)	**1.230** (0.500–4.540)	**1.000** (0.600–2.500)	**1.050** (0.200–10.900)

Esophageal cancer (*n* = 49) Total group	Median (range) *p* (EC versus healthy controls)	**1.277** (0.108–3.106) 0.044^*∗*^	**0.443** (0.049–1.516) 0.031^*∗*^	**2.220** (0.500–65.060) 0.003^*∗*^	**1.200** (0.400–8.100) 0.567	**6.500** (0.200–217.600) <0.001^*∗*^

Adenocarcinoma of esophagus (AC) (*n* = 24)	Median (range) *p* (AC versus healthy controls)	**1.118** (0.108–2.697) 0.216	**0.403** (0.049–1.516) 0.026^*∗*^	**1.955** (0.500–46.550) 0.034^*∗*^	**0.800** (0.400–2.200) 0.154	**2.800** (0.200–46.800) 0.031^*∗*^

Squamous cell cancer of esophagus (ESCC) (*n* = 25)	Median (range) *p* (ESCC versus healthy controls) *p* (AC versus ESCC)	**1.501** (0.380–3.106) 0.030^*∗*^ 0.562	**0.534** (0.076–1.466) 0.146 0.168	**2.250** (0.500–65.060) 0.013^*∗*^ 0.904	**1.400** (0.500–8.100) 0.006^*∗*^ 0.003^*∗*^	**14.900** (0.200–217.600) <0.001^*∗*^ 0.008^*∗*^

^*∗*^Statistically significant when *p* < 0.05.

**Table 3:** Diagnostic criteria for chemokine CXCL12 and its receptor (CXCR4), classical tumor markers (CEA and SCC-Ag), and C-reactive protein (CRP) levels in esophageal cancer (EC) patients.

	Diagnostic sensitivity	Diagnostic specificity	Positive predictive value	Negative predictive value	Diagnostic accuracy
CXCL12	47	80	79	48	59
CXCR4	80	57	75	63	71
CEA	22	93	85	42	49
SCC-Ag	14	97	88	41	46
CRP	57	97	97	58	72
CXCL12 + CXCR4	94	37	71	79	72
CEA + SCC-Ag	33	90	84	45	54
CXCL12 + CRP	73	80	86	65	76
CXCL12 + CEA	59	77	81	53	66
CXCL12 + SCC-Ag	59	77	81	53	66
CXCR4 + CRP	92	53	76	80	77
CXCR4 + CEA	84	53	75	67	72
CXCR4 + SCC-Ag	82	53	74	64	71
